# Generalized net modelling of the male urinary system with prostate-related outflow obstruction

**DOI:** 10.3389/fnetp.2026.1888801

**Published:** 2026-08-25

**Authors:** Martin Lubich, Krassimir Atanassov, Peter Vassilev, Vassia Atanassova, Chavdar Slavov, Elenko Popov, Lyudmila Todorova

**Affiliations:** 1 University Hospital “Sofiamed”, Sofia, Bulgaria; 2 Department of Bioinformatics and Mathematical Modelling, Institute of Biophysics and Biomedical Engineering, Bulgarian Academy of Sciences, Sofia, Bulgaria; 3 Faculty of Medicine, Acibadem City Clinic Tokuda Univesity Hospital, Sofia, Bulgaria; 4 Medical University Sofia, Sofia, Bulgaria; 5 Department of Urology and Andrology, University Hospital Tsaritsa Yoanna-ISUL, Sofia, Bulgaria

**Keywords:** generalized nets, human body, mathematical modelling, network physiology, prostate gland, urinary system

## Abstract

The present paper proposes a generalized net model of the male urinary system that reflects the principal anatomical and functional relations among the kidneys, ureters, urinary bladder, urethra, nervous system, muscle system, and prostate gland, both in norm and pathology. Particular attention is given to the role of the prostate gland and to changes in urinary flow and bladder emptying associated with prostate-related outflow obstruction. By representing material and informational flows and their interactions across several physiological subsystems, the model is conceptually aligned with the perspective of network physiology. The proposed framework provides a formal basis for the qualitative comparison of normal and pathological states and for future extension, parameterization, scenario-based simulation, and clinical validation.

## 1 Introduction

The functioning of the male urinary system depends on coordinated interactions among the kidneys, ureters, urinary bladder, urethra, nervous system, muscle system, and prostate gland. Prostate-related bladder outlet obstruction may disturb urinary flow and bladder emptying and may consequently affect several interconnected physiological processes.

Generalized Nets (GNs) have already been successfully applied to the modelling of parallel, concurrent, and interdependent processes in various biological and medical systems. Their capacity to formally represent state transitions, conditions, information carriers (tokens), and temporal characteristics allows them to describe complex networked physiological mechanisms in a precise and flexible way. Until now, GNs have been used for the formal description of different systems of the human body; however, their application to the detailed modelling of the male urinary system, with special attention to the influence of the prostate gland, has not been sufficiently investigated.

The present paper proposes such a model by representing the principal anatomical and functional relations among the kidneys, ureters, urinary bladder, urethra, prostate gland, nervous system, and muscle system. The model describes renal blood processing, urine transport, bladder filling and emptying, and the modification of urinary outflow under prostate-related obstruction. It provides a formal basis for the qualitative comparison of normal and pathological states and for future simulation studies, model extension, parameterization, and analysis.

## 2 Materials and methods

This section presents the physiological basis adopted for the model and describes the generalized-net formalism and construction procedure used to represent the principal anatomical structures, physiological processes, and interactions of the male urinary system.

### 2.1 Physiological basis of the model

The kidneys are two bean-shaped organs that play a central role in removing metabolic waste and surplus water from the body, thereby helping to regulate fluid volume and electrolyte homeostasis. This regulatory function depends mainly on the filtration of blood. Blood reaches the kidneys through the renal arteries, which originate from the abdominal aorta and subsequently branch into progressively smaller vessels. These vascular branches ultimately give rise to the glomerular capillary plexus. The nephron is the functional unit of the kidney, and its glomerulus is the capillary structure in which blood filtration begins. Blood enters the glomerulus through the afferent arteriole and leaves through the efferent arteriole. Under normal physiological conditions, the kidneys receive approximately 20– 25% of the cardiac output, (Kanwar and Venkatachalam, 1991). This substantial blood supply supports filtration across the large number of nephrons in the kidneys. Each kidney contains approximately one–1.5 million nephrons, although the number varies considerably among individuals.

Glomerular filtration, also referred to as renal ultrafiltration, is driven primarily by the hydrostatic pressure generated within the glomerular capillaries. This pressure drives water and small solutes across the glomerular filtration barrier, which consists of the fenestrated capillary endothelium, the glomerular basement membrane, and the filtration slits between podocyte foot processes. During this process, filterable blood components, including water and small solutes such as nitrogenous waste products, enter Bowman’s space, whereas nonfilterable components, such as blood cells and most plasma proteins, remain within the vascular compartment and leave the glomerulus through the efferent arteriole to rejoin the bloodstream (Miner, 2012). The ability of a substance to cross the filtration barrier is determined by factors including molecular size and electrical charge. The negative charge of components of the glomerular filtration barrier restricts the passage of negatively charged plasma proteins (Shoskes and McMahon, 2012). The resulting fluid constitutes the glomerular filtrate and subsequently passes from Bowman’s space into the renal tubule.

Glomerular Filtration Rate (GFR) refers to the volume of fluid filtered by the kidneys per unit time. It is mainly determined by three physiological factors (Mundy and Fitzpatrick, 2010, pp. 57–82): (1) the difference in hydrostatic and oncotic pressure; (2) the renal plasma flow; and (3) the permeability of the glomerular membrane. In a healthy young adult male, the GFR is approximately 180 L/24 h, which corresponds to about 125 mL/min (Kaufman et al., 2019).

In a typical adult, the Blood Volume (BV) is around 5 L. Plasma accounts for nearly 55% of the BV, giving a plasma volume of approximately 3 L. Renal Blood Flow (RBF) is about 1.1 L/min. Accordingly, Renal Plasma Flow (RPF) can be estimated as: 0.55×1.1 l/min=605 ml/min . This means that for every 605 mL that enters the glomeruli each minute through the afferent arterioles, about 125 mL, or nearly 20% , is filtered. The remaining 480 mL continues through the efferent arterioles and returns to the bloodstream.

Evaluating GFR is a key requirement for assessing renal function. The GFR is determined by the water permeability of the filtration membrane, the available filtration surface area, and the Net Filtration Pressure (NFP). Therefore, the filtration rate may be expressed as the product of water permeability, filtration area, and NFP. Since direct measurement of the total capillary bed surface area is highly difficult, the glomerular filtration coefficient $K_{f}$ is commonly used instead. This coefficient incorporates both the water permeability of the membrane and the effective filtration area.

NFP represents the algebraic sum of hydrostatic and osmotic pressures, including protein-oncotic or colloid-osmotic pressures. Four opposing pressures are involved in this balance: two hydrostatic pressures and two oncotic pressures. GFR is not fixed; rather, it varies substantially under different physiological and pathological conditions. In many diseases, the reduction in GFR (Aboumarzouk, 2019, pp. 107–155) is caused not primarily by changes in these pressure-related parameters, but by a loss of functioning nephrons, which reduces the total glomerular filtration surface area and, consequently, in $K_{f}$ .

In the proposed model, nephron-level processes, including glomerular filtration, tubular reabsorption, tubular secretion, and urine concentration, are aggregated within each kidney transition. Thus, the kidney transitions represent the overall transformation of incoming renal blood flow into blood returned to the cardiovascular system and urine conveyed toward the urinary bladder, rather than each intrarenal process separately.

The Prostate Gland (PG) and the urinary system are closely related anatomically and functionally. The PG is part of the male reproductive system that produces seminal fluid, which supports the mobility of spermatozoa and participates in the act of ejaculation. In young adult men, the prostate gland typically has a volume of approximately 20 mL (Berry et al., 1984). Prostate volume may increase with advancing age, particularly in association with the development of benign prostatic hyperplasia (BPH), although the extent and rate of enlargement vary considerably among individuals (Berry et al., 1984; Rhodes et al., 1999; Loeb et al., 2009). A prostate volume greater than approximately 30 mL is commonly used as a clinical threshold for prostatic enlargement (European Association of Urology, 2026).

The PG is situated beneath the urinary bladder and in front of the rectum. It surrounds the proximal portion of the urethra, thereby establishing its direct anatomical connection with the urinary tract, which comprises the kidneys, ureters, urinary bladder, and urethra. In BPH, enlargement of the prostate compresses the urethra and impedes the normal flow of urine. This may lead to a weak urinary stream, difficulty initiating urination, nocturia, a sensation of incomplete bladder emptying, double voiding (need to void again shortly after urination), and the need to apply intra-abdominal pressure during urination. These symptoms are collectively referred to as Lower Urinary Tract Symptoms.

### 2.2 Generalized nets and model construction

Generalized Nets [GNs, see (Atanassov, 1991; Atanassov, 2007; Alexieva et al., 2007; Zoteva and Angelova, 2021)] provide a formal framework for modelling parallel, concurrent, and interacting processes and represent an extension of Petri nets and their modifications. GNs have been applied in medicine and to the formal description of different systems of the human body.

The proposed model was constructed analytically using the formal apparatus of generalized nets. No patient-level dataset, automated model-generation tool, or proprietary software was used in its construction. The principal anatomical entities, physiological processes, and interactions included in the model were identified on the basis of the cited medical literature and authors’ clinical expertise.

In constructing the model, organs and functional subsystems were represented as GN transitions, while the intermediate physiological states and points of interaction were represented as places. Material flows, such as blood and urine, and informational flows, such as signals exchanged between the bladder, nervous system, and muscle system, were represented by tokens with corresponding characteristics. The transition predicates describe the conditions under which the tokens move between places, merge, or split.

The formal specification was examined for internal consistency with respect to the correspondence between input and output places, the continuity of token pathways, the use of the declared token types, the consistency of transition predicates, and the symmetry of the left- and right-kidney representations. The resulting model structure and its formal specification are presented in Section 3.

## 3 Model structure and formal specification

**FIGURE 1:**
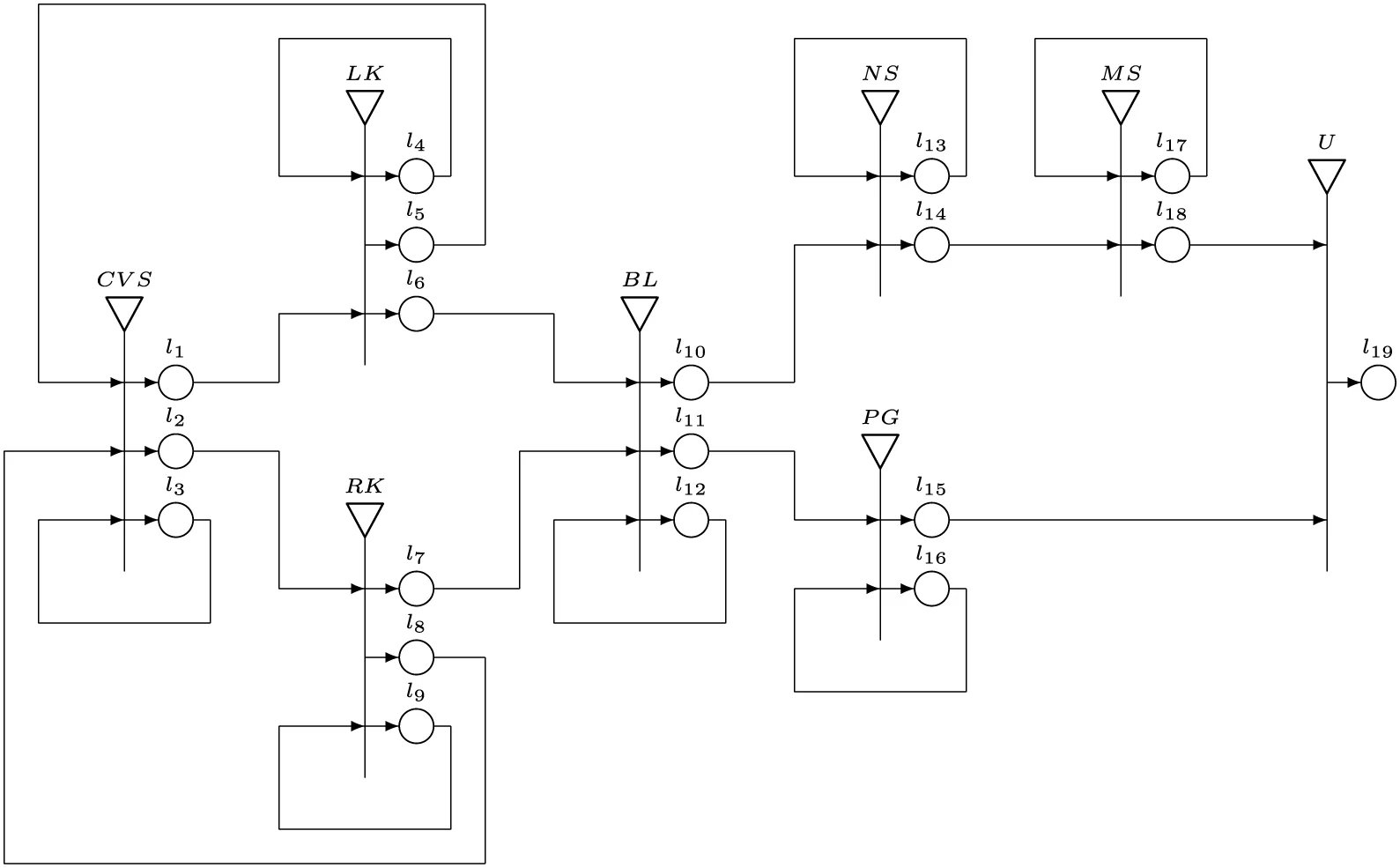
GN model of the male urinary system with prostate-related outflow obstruction.

The GN from Figure 1 describes the male urinary system with prostate-related outflow obstruction. The GN contains 8 transitions, 19 places and 17 types of tokens.

The following human body systems and organs are represented by the following GN-transitions:

CVS – Cardiovascular System;

LK – Left Kidney;

RK – Right Kidney;

BL – Bladder;

NS – Nervous System;

PG – Prostate Gland;

MS – Muscle System;

U – Urethra.

In our research, the CVS, NS, MS and organs (both kidneys, prostate gland, two ureters and urethra) are given in a simplified form.

The different token types, their meaning and initial places in the GN are the following:

β – monitors and reports the current status of the CVS (place $l_{3}$ );

λ – monitors and reports the current status of the LK (place $l_{4}$ );

ρ – monitors and reports the current status of the RK (place $l_{9}$ );

ω – monitors and reports the current status of the bladder, including the quantity of urine contained in it and, in the pathological case, the residual urine remaining after incomplete emptying (place $l_{12}$ );

ν – monitors and reports the current status of the NS (place $l_{13}$ );

π – monitors and reports the current status of the PG (place $l_{16}$ );

μ – monitors and reports the current status of the MS (place $l_{17}$ );

$\alpha_{L}$ , $\alpha_{R}$ – blood to the left/right kidney, quantity (places $l_{1},l_{2}$ , respectively);

$\gamma_{L}$ , $\gamma_{R}$ – urine produced by the left/right kidney and conveyed toward the urinary bladder (places $l_{6},l_{7}$ , respectively);

$\delta_{L}$ , $\delta_{R}$ – blood components returned from the left/right kidney to the CVS (places $l_{5},l_{8}$ , respectively);

ζ – a signal from bladder to NS for contraction (place $l_{10}$ );

υ – urine from bladder to the urethra (before the PG) (place $l_{11}$ );

σ – a signal from NS to MS for contraction (place $l_{14}$ );

τ – command for detrusor contraction (place $l_{18}$ ).

Before proceeding with the formal description of the transitions in the generalized net model, we will just outline the essence of the proposed model in a few sentences. Blood from the cardiovascular system (represented by place $l_{3}$ ) enters the left $\left(l_{4}\right)$ and right $\left(l_{9}\right)$ kidneys and, after being purified, returns to the cardiovascular system, and the substances excreted from it through the ureters go to the bladder $\left(l_{12}\right)$ . When the bladder is full, a signal is sent from it through the nervous system $\left(l_{13}\right)$ to the bladder muscles $\left(l_{18}\right)$ , which causes urine to be pushed out of the bladder into the urethra. When the prostate gland $\left(l_{16}\right)$ is functioning normally, urine passes through the prostatic urethra without significant obstruction. In the pathological branch of the model, prostate-related bladder outlet obstruction reduces urinary outflow and may result in incomplete bladder emptying and residual urine.

To simplify the model, we assume that the blood is generated at place $l_{3}$ .

Further we will provide a formal description of the transitions in the GN model.

The first transition, CVS , has the form:

$$CVS=\langle \left\{l_{3},l_{5},l_{8}\right\},\left\{l_{1},l_{2},l_{3}\right\},\begin{array}{cccc} & l_{1} & l_{2} & l_{3}\\ l_{3} & true & true & true\\ l_{5} & false & false & true\\ l_{8} & false & false & true \end{array}\rangle .$$

At each time-step, the token $\delta_{L}$ from place $l_{5}$ —and $\delta_{R}$ from place $l_{8}$ , respectively—enters place $l_{3}$ and merges with token β that obtains the characteristics *“current status of the CVS”*.

The token β splits to three tokens–the same token β without a new characteristic and tokens $\alpha_{L}$ and $\alpha_{R}$ that enter places $l_{1}$ and $l_{2}$ , respectively, with equal characteristics: *“blood, perfusion volume”*.

The next two transitions, LK and RK , have analogous forms. Each kidney transition represents the renal processes at an aggregated organ level rather than modelling the individual nephron stages. More specifically, transition LK representing the left kidney, has the form:

$$LK=\langle \left\{l_{1},l_{4}\right\},\left\{l_{4},l_{5},l_{6}\right\},\begin{array}{cccc} & l_{4} & l_{5} & l_{6}\\ l_{1} & true & false & false\\ l_{4} & true & true & true \end{array}\rangle .$$

At each time-step, the token $\alpha_{L}$ from place $l_{1}$ enters place $l_{4}$ and merges with token λ that obtains the characteristic *“current status of the LK”*.

The token λ splits into three tokens–the same token λ without a new characteristic and tokens $\delta_{L}$ and $\gamma_{L}$ that enter places $l_{5}$ and $l_{6}$ , respectively, with characteristics:

- *“blood returned from the LK to the CVS; quantity”*, and
- *“urine produced by the LK and conveyed toward the left ureter; quantity”.*

Similarly, the transition RK representing the right kidney, has the form:

$$RK=\langle \left\{l_{2},l_{9}\right\},\left\{l_{7},l_{8},l_{9}\right\},\begin{array}{cccc} & l_{7} & l_{8} & l_{9}\\ l_{2} & \text{false} & \text{false} & true\\ l_{9} & true & \text{true} & \text{true} \end{array}\rangle .$$

At each time-step, the token $\alpha_{\text{R}}$ from place $l_{2}$ enters place $l_{9}$ and unites with token ρ that obtains the characteristic *“current status of the RK”*.

The token ρ splits into three tokens–the same token ρ without a new characteristic and tokens $\delta_{\text{R}}$ and $\gamma_{\text{R}}$ that enter places $l_{8}$ and $l_{7}$ , respectively, with characteristics:

- *“blood returned from the RK to the CVS; quantity”*, and
- *“urine produced by the RK and conveyed toward the right ureter; quantity”.*

The subsequent transition that describes the bladder, BL , has the form:

$$BL=\langle \left\{l_{6},l_{7},l_{12}\right\},\left\{l_{10},l_{11},l_{12}\right\},\begin{array}{cccc} & l_{10} & l_{11} & l_{12}\\ l_{6} & false & false & true\\ l_{7} & false & false & true\\ l_{12} & W_{12,10} & W_{12,11} & true \end{array}\rangle ,$$

where $W_{12,10}=W_{12,11}=$ “there is enough quantity of urine in the bladder”.

At each time-step, the token $\gamma_{L}$ from place $l_{6}$ —and $\gamma_{R}$ from place $l_{7}$ , respectively—enters place $l_{12}$ and unites with token ω that obtains the characteristic *“current status of the BL”*.

When the predicates $W_{12,10}$ and $W_{12,11}$ become *true*, the token ω splits to three tokens–the same token ω without a new characteristic, and the tokens ζ and υ . Token ζ enters place $l_{10}$ with one of the two characteristics:

- *“signal from stretch receptors to NS”,* if there is no problem with the PG, or
- *“increased frequency of bladder emptying impulses to NS”*, if there is a problem with the PG.

Token υ enters place $l_{11}$ , with the characteristic *“urine passes from the bladder to the urethra”*.

The part of the nervous system involved in the described process is represented in the GN model by transition NS , and has the form:

$$NS=\langle \left\{l_{10},l_{13}\right\}\left\{l_{13},l_{14}\right\},\begin{array}{ccc} & l_{13} & l_{14}\\ l_{10} & true & false\\ l_{13} & true & W_{13,14} \end{array}\rangle ,$$

where $W_{13,14}=$ “there is a signal from bladder that there is enough quantity of urine in it”.

When the token ζ from place $l_{10}$ enters place $l_{13}$ , it unites with token ν that obtains the characteristic *“current status of the NS”*.

When the predicate $W_{13,14}$ becomes *true*, the token ν splits to two tokens–the same token ν without a new characteristic and a token σ that enters place $l_{14}$ with a characteristic: *“signal to MS for contraction”*.

The GN transition PG , that corresponds to the prostate gland, has the following form in the model:

$$PG=\langle \left\{l_{11},l_{16}\right\},\left\{l_{15},l_{16}\right\},\begin{array}{ccc} & l_{15} & l_{16}\\ l_{11} & true & false\\ l_{16} & false & true \end{array}\rangle .$$

When there is a token υ in place $l_{11}$ , it enters place $l_{15}$ with one of the following two characteristics:

- *“the urine from the bladder is emptied without prostate-related obstruction”*, if there is no problem with the PG, and
- *“urinary outflow is reduced, resulting in incomplete bladder emptying and residual urine”*, if there is prostate-related bladder outlet obstruction.

The token υ represents the quantity of urine leaving the bladder. In the pathological case, the residual quantity of urine is reflected in the updated characteristic of the bladder-status token ω , which remains in place $l_{12}$ .

At each time-step, the token π from place $l_{16}$ obtains the characteristic *“current status of the PG”*.

The next transition, MS , corresponds to that part of the muscle system which is involved in the modelled process, and it has the form:

$$MS=\langle \left\{l_{14},l_{17}\right\},\left\{l_{17},l_{18}\right\},\begin{array}{ccc} & l_{17} & l_{18}\\ l_{14} & true & false\\ l_{17} & true & W_{17,18} \end{array}\rangle ,$$

where $W_{17,18}=$ “contraction of the detrusor muscle is necessary”.

When the token σ from place $l_{14}$ enters place $l_{17}$ , it unites with token μ that obtains the characteristic *“current status of the MS”*.

When the predicate $W_{17,18}$ becomes *true*, the token μ splits to two tokens–the same token μ without a new characteristic and a token τ that enters place $l_{18}$ with a characteristic: *“signal from MS for contraction”*.

Finally, transition U has the form:

$$U=\langle \left\{l_{15},l_{18}\right\},\left\{l_{19}\right\},\begin{array}{cc} & l_{19}\\ l_{15} & W_{15\text{, }19}\\ l_{18} & true \end{array}\rangle ,$$

where $W_{15\text{, }19}=$ *“there is a contraction of the detrusor muscle”*.

The token υ from place $l_{15}$ and the token τ from place $l_{18}$ are united in place $l_{19}$ in a token υ with a characteristic: *“final urine; quantity”*.

## 4 Discussion

The GN model of the male urinary system represents the logical connections between this system and some other systems of the human body. It It extends earlier generalized net research on the human body and complements more focused models concerning nephrological diseases, prostate cancer development, and the evaluation of lower urinary tract symptoms (Lubich et al., 2023; Popov et al., 2023; Mirinchev et al., 2026). Compared with the model presented in Atanassov et al. (2008), the present construction provides a more detailed representation of the male urinary system by explicitly including the role of the prostate gland and the effects of prostate-related bladder outlet obstruction on urinary flow. As the systems and organs involved are presented in a simplified form, the model allows further sophistication in future, as well as inclusion as a subset of other generalized net models of the human body (see Atanassov et al., 2008). The proposed model may provide a conceptual basis for educational scenarios and, after appropriate parameterization and clinical validation, for the future development of medical decision-support applications.

The model is intentionally qualitative and operates at an aggregated organ-system level. It does not represent the detailed intrarenal, urodynamic, neurological, and muscular mechanisms involved in urine formation and voiding, nor does it distinguish among different degrees of prostate-related obstruction. Consequently, the pathological branch captures only the principal effects of prostate-related obstruction and does not encompass the full heterogeneity of benign prostatic hyperplasia and male lower urinary tract symptoms.

The present study proposes a conceptual GN model and presents its internal logical structure; it does not perform clinical or data-driven validation. Such validation would require temporally aligned measurements of renal function, bladder filling and emptying, neural signalling, detrusor activity, prostate-related obstruction, and urine flow in individual patients. The absence of such validation constitutes an important limitation of the present work. Despite this limitation, the proposed model provides a coherent formal representation of the principal material and informational flows involved in the male urinary system, with explicit consideration of the role of the prostate gland and prostate-related bladder outlet obstruction. Future development may further include clinically defined degrees of obstruction, thereby enabling scenario-based simulation and comparison with patient observations.

## Data Availability

The original contributions presented in the study are included in the article/supplementary material, further inquiries can be directed to the corresponding author.
